# Development of a Multiplex HIV/TB Diagnostic Assay Based on the Microarray Technology

**DOI:** 10.3390/bios13090894

**Published:** 2023-09-20

**Authors:** Kanyane Malatji, Advaita Singh, Christina Thobakgale, Kabamba Alexandre

**Affiliations:** 1 Array Technology Laboratory, Synthetic Biology and Precision Medicine Centre: Next Generation Health Cluster, Council for Scientific and Industrial Research, Brummeria, Pretoria 0001, South Africakabamba.alexandre@gmail.com (K.A.); 2 Department of Virology, School of Pathology, Faculty of Health Sciences, University of the Witwatersrand, Braamfontein, Johannesburg 2000, South Africa; christina.thobakgale@wits.ac.za; 3 Future Production: Chemicals Cluster, Council for Scientific and Industrial Research, Brummeria, Pretoria 0001, South Africa; 4 Centre for HIV and STIs, National Institute for Communicable Diseases, Sandringham, Johannesburg 2192, South Africa

**Keywords:** HIV-1 p24, *M.tb* CFP10, *M.tb* ESAT6, *M.tb* pstS1, multiplex microarray, diagnosis, antibody, antigen

## Abstract

Currently there are diagnostic tests available for human immunodeficiency virus (HIV) and tuberculosis (TB); however, they are still diagnosed separately, which can delay treatment in cases of co-infection. Here we report on a multiplex microarray technology for the detection of HIV and TB antibodies using p24 as well as TB CFP10, ESAT6 and pstS1 antigens on epoxy-silane slides. To test this technology for antigen–antibody interactions, immobilized antigens were exposed to human sera spiked with physiological concentrations of primary antibodies, followed by secondary antibodies conjugated to a fluorescent reporter. HIV and TB antibodies were captured with no cross-reactivity observed. The sensitivity of the slides was compared to that of high-binding plates. We found that the slides were more sensitive, with the detection limit being 0.000954 µg/mL compared to 4.637 µg/mL for the plates. Furthermore, stability studies revealed that the immobilized antigens could be stored dry for at least 90 days and remained stable across all pH and temperatures assessed, with pH 7.4 and 25 °C being optimal. The data collectively suggested that the HIV/TB multiplex detection technology we developed has the potential for use to diagnose HIV and TB co-infection, and thus can be developed further for the purpose.

## 1. Introduction

An estimated 38.0 million people were living with HIV/AIDS globally with the annual number of deaths standing at 970,000 in 2021, and 25% were co-infected with *Mycobacterium tuberculosis* (*M.tb*), the causative agent of TB [1]. Albeit that there are diagnostic tests currently available for HIV and TB, the diagnosis of these two diseases is still performed separately and this can delay treatment in co-infected individuals, particularly because active TB diagnosis can take a long time [2,3]. With HIV, there is a rapid test for the detection of p24, in addition to the PCR test. However, the currently existing antibody test for TB suffers from low sensitivity and specificity, especially in HIV-infected individuals [4,5]. Furthermore, there is also a polymerase chain reaction (PCR) test available for TB detection, although this test cannot distinguish between active and latent TB, thus requiring the culturing of the bacteria to confirm the active infection, which can take time [6,7,8]. Therefore, there is a need for alternative diagnostic technology that will allow for the simultaneous detection of HIV and TB more quickly, as well as the ability to distinguish between active and latent TB. 

Microarray technology refers to the miniaturization of multiple assays on one small plate or slide [9,10,11,12]. There are different types of microarrays, which include analytical and functional microarrays [11]. An example of an analytical microarray is the antibody array, which involves protein detection and concentration determination after antibody capture using direct protein labelling [11,13,14]. The functional microarray is built using purified proteins to enable the study of various biochemical properties such as protein–protein interactions, protein binding activities, protein–peptide interactions and protein–lipid interactions, to name a few [15,16,17,18]. Protein microarray technology allows for the characterization of hundreds of thousands of proteins in a highly parallel and high-throughput manner [19,20,21]. Immunoassays take advantage of highly specific antigen–antibody recognition to build a protein detection system, whereas the microarray enables a rapid parallel and multiplex detection, often requiring a small amount of sample [22,23]. The immunoassay on microarrays can be very sensitive and specific, thus making it very reliable when studying or determining antigen–antibody interactions such as occurs in diagnostics [24,25,26].

Epoxy-coated slide chemistry is one of the commonly used systems for protein microarray, as they are stable under various conditions and are reactive with several nucleophilic groups to form strong bonds with minimal chemical modification of the protein [27]. For example, epoxy-coated glass slides have been used in cancer studies as well as in the detection of antibodies against SARS-CoV-2 for disease diagnostic purposes [22,28]. The interaction between the coated slide and proteins occurs through fast adsorption followed by intramolecular chemical attachment promoted by the epoxy groups on the glass surface [27]. The part of proteins that reacts with epoxy on the solid surfaces is mostly the amine group, such as found in lysine residues which are present on the exterior of proteins, forming stable amine bonds with the surface [29,30,31]. During the coupling process, proteins are dissolved in a low ionic-strength buffer before being passed through the coated solid surface. The immobilization efficiency depends on parameters such as pH, concentration and reaction time [27,30]. 

**Scheme 1:**
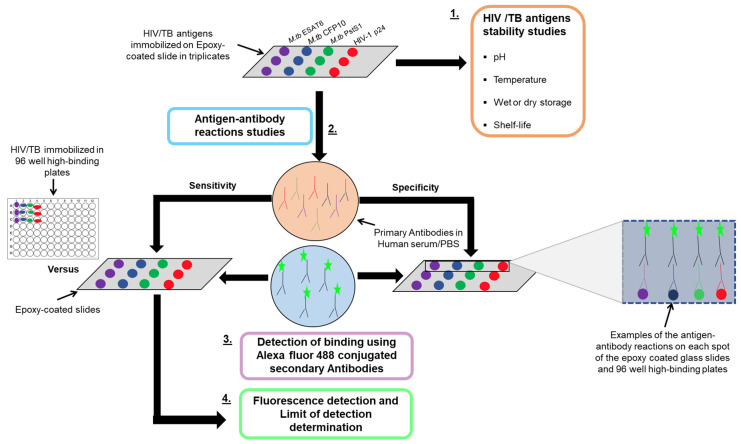
A schematic diagram showing the representation of the research strategy used to develop the HIV and TB multiplex microarray technology. The strategy started with stability studies of the immobilized antigens followed by sensitivity and specificity studies.

In this study, we developed a multiplex microarray technology on a 2.5 × 7.6 cm epoxy-coated glass slide for the detection of HIV and TB co-infection using HIV-1 p24 antigen as well as *M.tb* CFP10, ESAT6 and pstS1 antigens, known to be markers of active TB [32,33,34,35], following the research strategy outlined in Scheme 1. We found that the immobilized antigens captured corresponding primary antibodies in human serum. In addition, we proved that this interaction was specific and with sensitivity higher than what would be obtained on high-binding ELISA plates. Furthermore, this diagnostic technology performed optimally at room temperature and pH 7.4, and this performance was maintained for at least 90 days during the shelf-life experiments. In all, these data indicate that our diagnostic technology is potentially a solution for a quicker method of simultaneously diagnosing HIV-1 and TB in cases of co-infection. 

## 2. Materials and Methods

### 2.1. Reagents

The HIV-1 p24 antigen and anti-p24 (Ms), anti-CFP10 (Rb) and anti-ESAT6 antibodies (Rb) were bought from Abcam (Cambridge, UK). The culture filtrate protein-10 (CFP10) antigen and early secretory antigenic target protein 6 (ESAT6) were obtained from BBI solutions (Caerphilly, UK). The phosphate-specific transport substrate binding protein-1 (pstS1) antigen and the pstS1 primary antibody (Ms) were bought from My BioSource (San Diego, CA, USA). The Goat (Gt) anti-Mouse (Ms)/anti-Rabbit (Rb) IgG superclonal secondary antibody, Alexa fluor 488 conjugates were obtained from Abcam (Cambridge, UK). The NEXTERION epoxy-silane-coated glass slides were bought from Schott laboratories (Mainz, Germany), while the Corning polystyrene high-binding costar assay plates were purchased from Corning Inc. (Corning, NY, USA). 

### 2.2. Immobilization of HIV-1 p24 as Well as M.tb CFP10 ESAT6 and pstS1 Antigens on Epoxy-Coated Glass Slides

Antigens (HIV-1 p24 as well as *M.tb* CFP10, ESAT6 and pstS1) were diluted to 500 µg/mL in 1 × phosphate buffered saline (PBS) pH 7.4 (Thermo Fisher Scientific, Waltham, MA, USA), with concentrations ranging from 500 µg/mL to 0.0762 µg/mL. The different concentrations of each antigen were spotted (1 µL) on the NEXTERION epoxy-silane-coated glass slides and incubated for 90 min at room temperature. The slides were thereafter washed three times for 5 min in 1 × PBS pH 7.4, followed by blocking for an hour in 1% PBS-bovine serum albumin (BSA) (Sigma Aldrich, Midland, MI, USA). This was followed by washing the slides three times in 1× PBS for 5 min to remove excess BSA. The slides were then allowed to dry at room temperature for 15 min and bright-field images of the slides were captured using a cytation3 multimode reader (Biotek Instruments, Winooski, VT, USA). To further profile and evaluate the immobilized antigens, the slides were incubated for an hour in a cocktail solution containing anti-p24, anti-CFP10, anti-ESAT6 and anti-pstS1 primary antibodies at a constant concentration of 3.906 µg/mL to determine if they could capture the antibodies in solution. Post-incubation with primary antibodies, the slides were washed and incubated for an hour in a cocktail solution of Goat (Gt) anti-Mouse (Ms)/anti-Rabbit (Rb) IgG superclonal secondary antibody, conjugated to Alexa fluor 488, at a constant concentration of 0.10 µg/mL. The slides were thereafter washed and imaged using a cytation3 multimode reader. 

### 2.3. Determination of the Optimal Buffer pH, Storage Condition, Temperature and Shelf-Life of Immobilized HIV and TB Antigens

To determine the optimal printing, washing and storage pH conditions for the HIV-1 p24 as well as *M.tb* CFP10, ESAT6 and pstS1 antigens on the epoxy-coated glass slides, five different pH conditions, adjusted using NaOH and HCl (Sigma Aldrich, Midland, MI, USA), ranging from 5.0 to 9.0 were evaluated. Jorgensen et al. showed that epoxy substrates exhibited high stability in pH conditions of 6.0, 7.5 and 9.0 [36], hence the choice of the aforementioned conditions. To achieve this, the antigens were spotted in triplicate at a contact concentration of 31.3 µg/mL in PBS at different pH, followed by immobilization on the glass slides as mentioned above. Washing steps were performed as above, also using the PBS at corresponding pH. 

The determination of the optimal pH was followed by the evaluation of whether the immobilized antigens should be stored dry or in buffer. This was done by storing the immobilized antigens dry or in PBS pH 7.4 for 24 h and then studying their interaction with primary antibodies. 

The next step was to determine the optimal temperature at which the immobilized antigens on the epoxy-coated glass slides could be stored. Yousefi et al. showed that epoxy slides had the best functionality and produced high fluorescence at different temperatures such as 4 °C, 25 °C and 40 °C [30], hence our choice of −20 °C, 4 °C, 15 °C, 25 °C and 37 °C for this study. However, we used 37 °C as it is the optimal human body temperature instead of the 40 °C that Yousefi et al. used. To achieve this, immobilized HIV and TB antigens were incubated dry at −20 °C, 4 °C, 15 °C, 25 °C and 37 °C for 24 h, followed by the addition of primary and secondary antibody, and subsequent detection of fluorescence.

Studies were also conducted to determine the immobilized antigens’ shelf-life. Jorgensen et al. showed that printed proteins for multiplexing can remain stable for up to three months [36]; therefore, we assessed the HIV and TB antigens on the slide for a period of three months. This was done by storing immobilized antigens slides dry for 1 day, 15, 30, 45, 60, 75 and 90 days at room temperature. All the slides for the different conditions were incubated in a cocktail of the HIV and TB recombinant primary antibodies at a constant concentration of 3.906 µg/mL. Following the incubation, the slides were washed and incubated in Gt anti-Ms/anti-Rb IgG superclonal secondary antibody, conjugated to Alexa fluor 488, at a constant concentration of 0.10 µg/mL for an hour at room temperature. After the incubation, the slides were washed, and fluorescence images were obtained using the cytation3 multimode reader.

### 2.4. In Vitro Specificity and Sensitivity of the Microarray-Based Detection System for HIV and M.tb Antibodies 

The immobilization of the HIV and TB antigens for both the slides and plates was carried out as mentioned above, except that for the high-binding black with clear bottom polystyrene 96-well plates (Sigma Aldrich, Midland, MI, USA) we used 5 µL of the antigens per well. This was followed by the addition of 4-fold serially diluted HIV and TB primary antibodies (for the slides, the series range was 250–0.000954 µg/mL, while for the plates it was 250–0.020 µg/mL) in PBS or human serum, and incubation for 2 h at room temperature. To determine that each antigen specifically reacted with its corresponding primary antibody in solution, an anti-p24, anti-CFP10, anti-ESAT6 and anti-pstS1 primary antibody cocktail solution, missing one of the antibodies at a time, was prepared and incubated with the immobilized antigens. Subsequently, the plate was washed three times with PBS for 5 min. Afterwards, 100 µL Gt anti-Ms/anti-Rb IgG superclonal secondary antibody conjugated to Alexa fluor 488 at a concentration of 0.10 µg/mL was added and incubated at room temperature for an hour. Post-incubation, the plate was washed as above, and fluorescence was measured using DTX 880 Multimode detector (Beckman Coulter Inc., Brea, CA, USA) at a wavelength of 485 nm. SoftMax Proversion 6.4 (Molecular Devices, LLC) was used for data acquisition and analysis of the plates. For the microarray slides, the detection was carried out using a cytation3 multimode reader, while ImageJ v1.54f software was used for analysis.

Specificity between the HIV and *M.tb* antigens and the anti-p24, anti-CFP10, anti-ESAT6 and anti-pstS1 recombinant primary antibodies was also investigated by immobilizing the antigens as aforementioned. The whole slide was then incubated with a solution containing one of the primary antibodies at a time at a constant concentration of 3.906 µg/mL. For example, one slide of the immobilized antigens was incubated for an hour in a solution containing anti-p24 antibody only, while other slides were incubated with either anti-CFP10, anti-ESAT6 or anti-pstS1 primary antibody. After the incubation, the slides were washed and incubated with the secondary antibodies at a constant concentration of 0.10 µg/mL for an hour at room temperature. Then excess secondary antibodies were washed and the slide was imaged using Cytation3 multimode reader.

### 2.5. Statistical Analysis 

Statistical analysis was performed using GraphPad Prism version 9.4.0 (GraphPad Software, Inc., California, USA). Data are presented as mean ± standard deviation of the mean, and each experiment was performed at least three times unless stated otherwise. Statistical significance was assessed using paired Student *t*-test when comparing two means of the same groups under separate scenarios. One-way analysis of variance (ANOVA) test was also used to assess the statistical significance between multiple groups under different scenarios using Tukey’s multiple comparison test. For all comparisons, *p* values less than 0.05 were considered statistically significant. 

## 3. Results

### 3.1. Immobilization of Antigens on Epoxy-Coated Glass Slides

**Figure 1:**
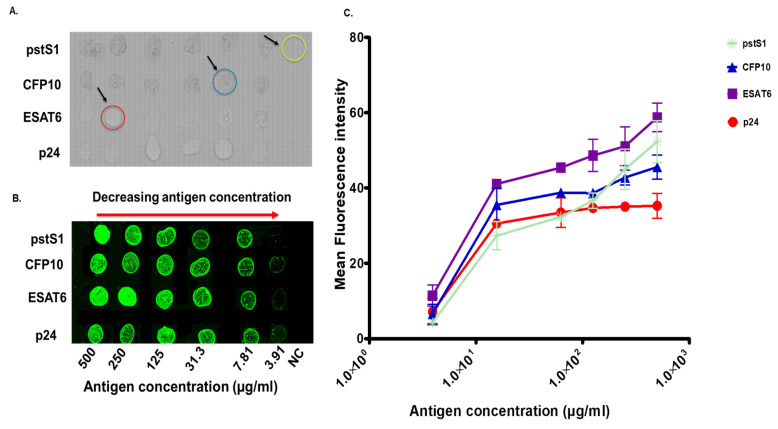
The immobilization of HIV-1 p24, M.tb CFP10, ESAT6 and pstS1 antigens on an epoxy-coated glass slide by covalent interactions. (**A**) The antigens were immobilized at different concentrations, PBS-only spots were used as negative control, and imaging was performed using bright-field microscopy. The antigen spots after washing and imaging are indicated by the red and blue circles, while yellow circles show the negative control. (**B**) The immobilized antigens were reacted with anti-p24, anti-CFP10, anti-ESAT6 and anti-pstS1 primary antibodies, and subsequently secondary antibodies conjugated to Alexa fluor 488 were added and the slide imaged with a cytation3 multimode reader with Gen5 2.08 software. (**C**) Mean fluorescence intensity of the spots was measured with ImageJ and plotted. The concentration at which the antigens were assessed is also shown. Error bars are means ± standard deviation (n = 3).

The immobilization of antigens as capture molecules on functionalized glass slides is a well-developed system for microarrays in diagnostic and drug discovery [37,38]. In this study, the microarray was developed by covalently immobilizing HIV-1 p24, *M.tb* CFP10, ESAT6 and pstS1 antigens on epoxy-coated glass slides. This was done to capture anti-p24, anti-CFP10, anti-ESAT6 and anti-pstS1 primary antibodies in solution. The immobilization was achieved by reacting the epoxy group on the glass surface with amine groups on the antigens to form a covalent amine bond. We then determined whether the immobilization of the TB and HIV antigens was successful, as well as the optimal conditions for this immobilization. We demonstrated that the spot where the antigens were printed was stable on the slide by means of bright-field imaging even after washing with PBS. This was shown on the bright-field image obtained using scanning microscopy by the presence of clear spots where antigens were printed (Figure 1A). Furthermore, these spots were observed for all concentrations (500–0.000228 µg/mL) and antigens printed, i.e., HIV-1 p24, as well as *M.tb* antigens CFP10, ESAT6 and pstS1, thus suggesting their interaction with the epoxy-coated glass slide. As a negative control, PBS was spotted in place of antigens, and, as expected, no spots were observed for PBS after visualization with the microscope (Figure 1A). To investigate whether the HIV p24 and *M.tb* CFP10, ESAT6 and pstS1 on the glass slide could react with their specific antibodies, the immobilized antigens were incubated with a cocktail containing anti-p24, anti-CFP10, anti-ESAT6 and anti-pstS1 primary antibodies. This was followed by the addition of a secondary antibody conjugated to Alexa fluor 488. The results showed that the coupled HIV and TB antigens could detect the primary antibodies even at the lowest concentrations of the antigens tested (3.91 µg/mL). This was indicated by the presence of green fluorescence from all the spots on the slide after imaging (Figure 1B). The mean fluorescence intensity of the spots on the image was obtained using the ImageJ software. From the graph in Figure 1C, we observed that the fluorescence intensity decreased with decreasing concentration of the antigens when the concentrations of the primary and secondary antibodies were kept constant at 3.906 µg/mL and 0.10 µg/mL, respectively. The antigen– antibody reaction that produced the highest fluorescence intensity, at the highest concentrations tested, was ESAT6 followed by pstS1. HIV-1 p24 antigen–antibody reaction showed the lowest fluorescence intensity while *M.tb* CFP10 was the second lowest fluorescence intensity relative to the other antigen–antibody reactions (Figure 1C). 

### 3.2. Investigation of the Stability of Immobilized Antigens on the Epoxy-Coated Glass Slides

**Figure 2:**
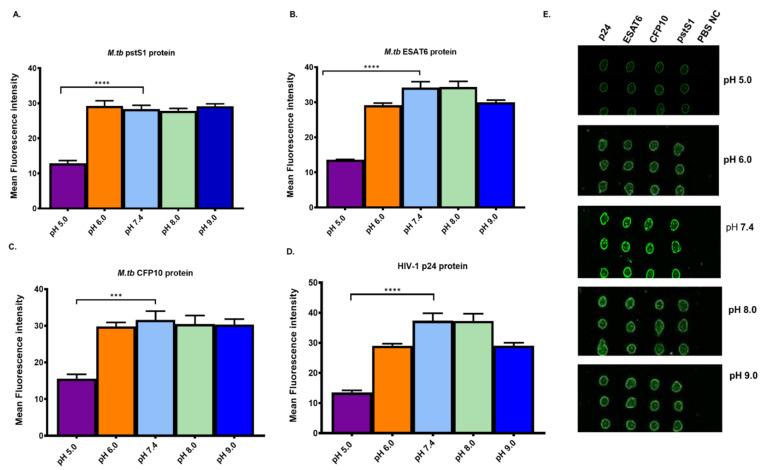
**Effect of different pH on the immobilization of antigens on epoxy-coated glass slides.** The antigens were printed and washed with PBS at different pH values. The bar graphs show fluorescence intensity obtained with *M.tb* pstS1 (**A**), CFP10 (**B**), ESAT6 (**C**) and HIV-1 p24 (**D**). Data represent mean ± standard deviation and error bars are standard deviation (n = 3). Asterisks signify statistical significance (*p* < 0.05) between the fluorescence intensity of the highest reading and the lowest. (**E**) shows representative images obtained at different pH (pH 5.0, 6.0, 7.4, 8.0 and 9.0).

After showing that the antigens were immobilized on the microarray slide and could interact with the antibodies, we determined the optimal storage pH, temperature and shelf-life of the immobilized antigen epoxy-coated slides. The pH studies were performed using PBS solutions at different pH values (pH 5.0, 6.0, 7.4, 8.0 and 9.0). The antigens were spotted at a concentration of 31.3 µg/mL in triplicate, as this was one of the concentrations where we observed a strong reaction with the primary antibodies, judging from the fluorescence obtained. After printing at the above-mentioned pH, the slide was blocked, followed by incubation with primary antibody at the corresponding pH. After addition of the secondary antibody, the slide was imaged. From the results obtained, the immobilized antigens (p24, ESAT6, CFP10 and pstS1) had similar interaction with primary antibody at all tested pH (pH 5.0, 6.0, 7.4, 8.0 and 9.0) (Figure 2). The highest fluorescence intensities observed at pH 6.0, 7.4, 8.0 and 9.0 were comparable, while at pH 5.0 there was 2-fold decrease in intensity, and this was statistically significant (*p* < 0.05) (Figure 2). Therefore, we concluded that the optimal pH range for the antigen–antibody reaction on our epoxy-coated slides was 6.0–9.0. 

**Figure 3:**
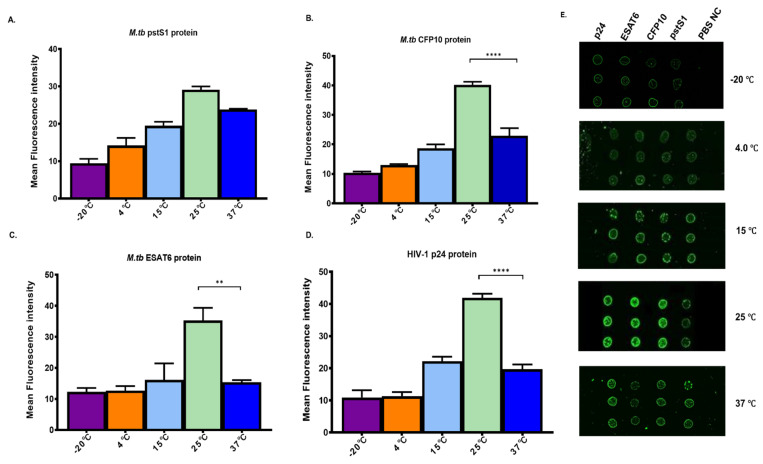
**Evaluation of the optimal storage temperature for the immobilized antigens**. The immobilized antigens were stored at different temperatures (−20 °C, 4 °C, 15 °C, 25 °C and 37 °C) for 24 h. The bar graphs of the fluorescence intensity obtained from *M.tb* pstS1 (**A**), CFP10 (**B**), ESAT6 (**C**) and HIV-1 p24 (**D**) are shown. Data represent mean ± standard deviation (n = 3) and asterisks indicate significant differences between the groups being compared. The slide images from which the bar graphs were plotted are shown in (**E**).

**Figure A1:**
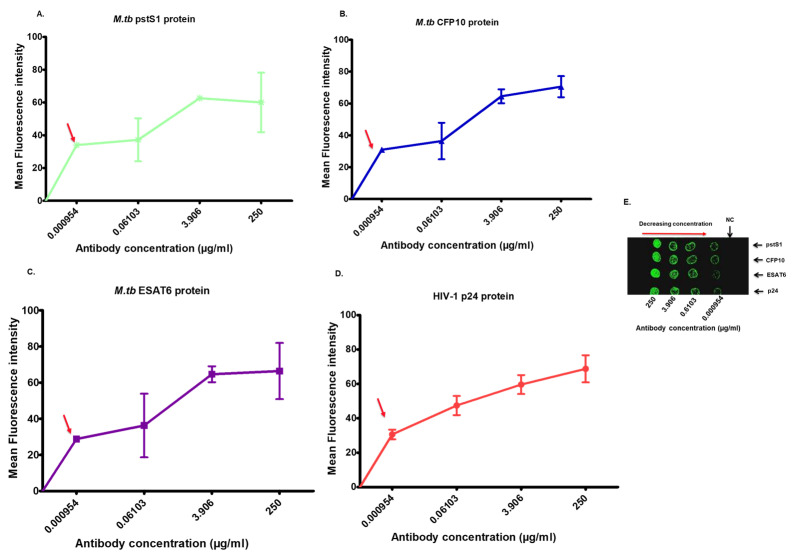
**Evaluation of sensitivity of epoxy-coated glass slides assay in PBS.** Primary antibodies were serially diluted in PBS while the antigen and secondary antibody concentrations were kept constant. Fluorescence intensity values for pstS1 (**A**), CFP10 (**B**), ESAT6 (**C**) and p24 (**D**) are presented as line graphs, and the limit of detection is indicated by the red arrow. Data represent mean ± standard deviation (n = 4). (**E**) A representative image of the fluorescence emitted on the microarray slide.

The optimal storage and reaction temperature for the immobilized antigens was also evaluated, as temperature can play an important role in the stability of proteins on functionalized microarray slides [30,36]. This study was performed by immobilizing the antigens on the epoxy-coated glass slides in triplicate and incubating them at different temperature conditions (−20 °C, 4 °C, 15 °C, 25 °C, 37 °C) for 24 h. This was followed by washing with PBS at pH 7.4, which was one of the optimal pH established previously. The antigens were reacted with a cocktail of their associated primary antibodies at the concentration of 3.906 µg/mL, and fluorescence was measured after the addition of secondary antibodies labelled with a fluorescence dye. The 3.906 µg/mL primary antibody concentration used was determined experimentally to be optimal (Appendix A, Figure A1). Based on the fluorescence intensities in Figure 3, the optimal temperature at which the p24, CFP10, ESAT6 and pstS1 antigens reacted with their antibodies was 25 °C, followed by 37 °C, 15 °C and 4 °C, while −20 °C had the lowest intensity. In fact, in general, the fluorescence intensity from slides stored at 25 °C was about 2-fold higher than the second-best storage temperature, and these differences were statistically significant (*p* < 0.05) for all antigens except pstS1 (Figure 3).

**Figure 4:**
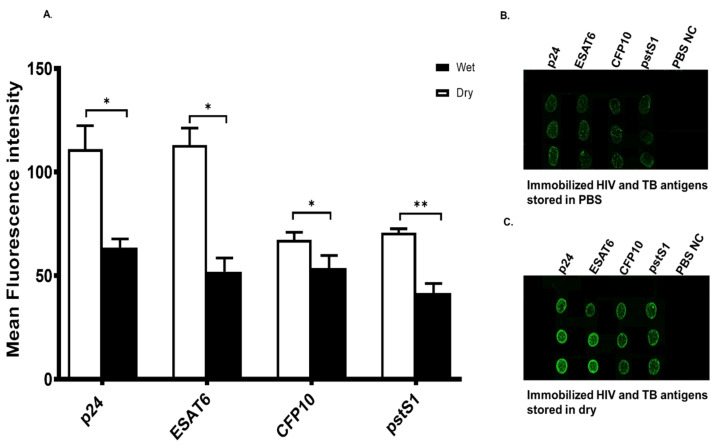
Evaluation of the stability in dry and wet storage conditions of the HIV and TB antigens immobilized on the epoxy-coated glass slide. The antigens were stored in PBS and dry at room temperature for 24 h. (**A**) The mean fluorescence of each spot was measured and plotted. Data represent mean ± standard deviation (n = 3) and asterisks show significant difference between groups. (**B**) and (**C**) representative PBS and dry storage images, respectively, used to plot the graph in (**A**).

After studying the stability of the immobilized antigens under different pH and temperature conditions, the next step was to determine whether the immobilized antigens on the epoxy glass slides should be stored dry or wet. We investigated this at pH 7.4 and room temperature, given that they were determined to be optimal. This was done by printing the HIV-p24 as well as *M.tb* CFP10, ESAT6 and pstS1 antigens on glass slides followed by 24 h incubation at room temperature in PBS pH 7.4, which represented the wet condition, or dry. From the results obtained, we observed fluorescence with both dry and wet slides (Figure 4). However, this intensity was lower for the antigens stored in PBS relative to the antigens stored dry (Figure 4B,C). In fact, the fluorescence intensity was decreased by 2-fold for HIV-1 p24 and ESAT6, while the decrease was about 1.5-fold for CFP10 and pstS1. All these decreases were statistically significant (*p* < 0.05). Therefore, storage in the dry proved to be the optimal condition. 

**Figure 5:**
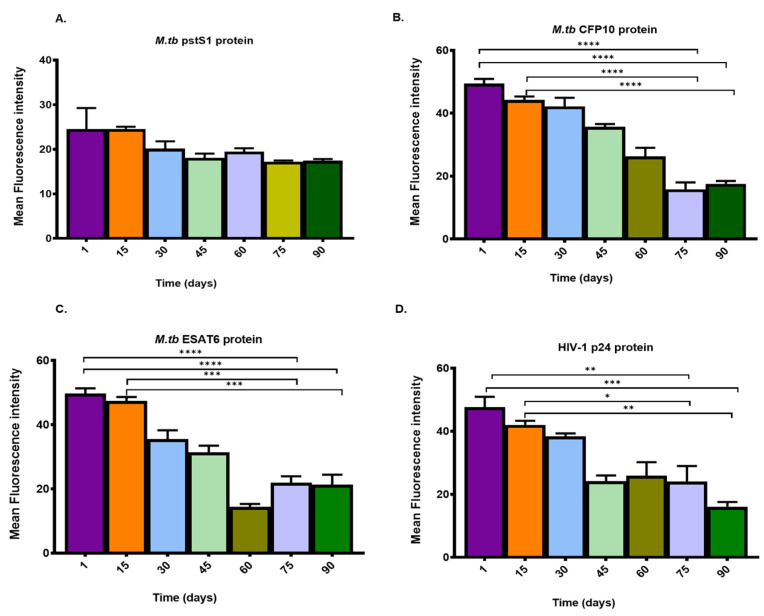
**Evaluation of the shelf-life of the immobilized HIV and TB antigens on the epoxy-coated glass slides**. The antigens were stored dry at room temperature from day 1 to day 90, followed by addition of primary and secondary antibodies, with fluorescence readings after every 15 days. The bar graphs show fluorescence intensity when *M.tb* pstS1 (**A**), CFP10 (**B**), ESAT6 (**C**) or HIV-1 p24 (**D**) were stored dry for 90 days. Data represent mean ± standard deviation (n = 2) and asterisks signify significant difference between the groups compared.

Lastly, we determined the shelf-life of the immobilized antigens on the epoxy-coated slides, in order to obtain an indication of the durability of the immobilized antigens on the slide. To achieve this, seven slides were coated with the HIV-1 p24 as well as *M.tb* CFP10, ESAT6 and pstS1 antigens at room temperature for an hour, washed and incubated dry (the optimal storage condition determined above) for different numbers of days. The incubation time points included day 1 to day 90 with fluorescence intensity readings at 15-day intervals. Fluorescence was detected on all antigens from day 1 through day 90 after the incubation of the immobilized antigens with primary and secondary antibodies (Figure 5). However, a decrease in the mean fluorescence intensity, as measured using the ImageJ software, was observed with longer periods of incubation, i.e., in most cases day 1 and day 15 yielded the highest fluorescence intensity while day 75 and day 90 had the lowest. In most cases, these differences were statistically significant (*p* < 0.05). In fact, there was a progressive and consistent decrease in fluorescence intensity emitted from the slides as the shelf-life moved toward day 90. However, it is important to note that even at day 90 there was still clear detection of all the antigens on the microarray slide, implying that our HIV/TB co-infection detection technology has the potential to last on the shelf for at least three months.

### 3.3. Comparison of the Antigen–Antibody Reactions between the Microarray Epoxy-Coated Slide Platform and the Protein Polystyrene High-Binding Plates 

**Figure 6:**
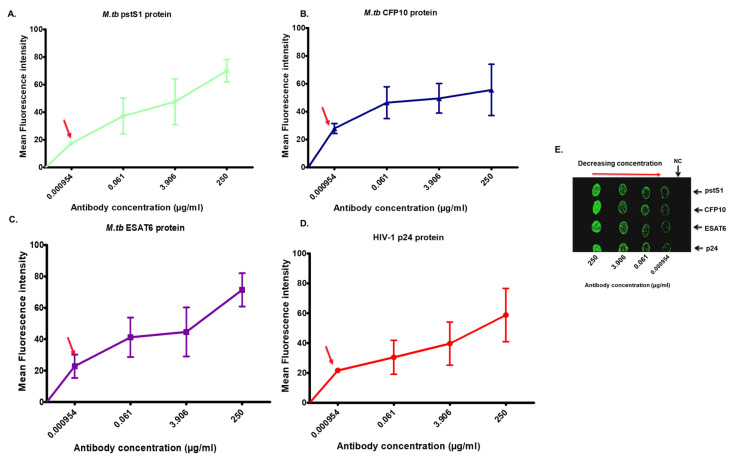
**Evaluation of the sensitivity of the assay using epoxy-coated glass slides.** Primary antibodies were diluted in human serum to mimic in vivo conditions where there are many other proteins present. The antigen and secondary antibody concentrations were kept constant. Fluorescence intensity values for pstS1 (**A**), CFP10 (**B**), ESAT6 (**C**) and p24 (**D**) are presented as line graphs for the different antibody concentrations, and the limit of detection is indicated by the red arrow. Data represent mean ± standard deviation (n = 4). (**E**) A representative image of the slide used to plot the graphs.

Next, we studied the sensitivity of the microarray-based multiplex technology. The assay was conducted by diluting antibodies in PBS or human serum and incubating them with the epoxy-coated slides containing immobilized antigens. The use of human serum was to determine how specific and sensitive the antigen–antibody reactions would be when the antibodies were in the presence of proteins that are commonly found in human blood. The concentrations evaluated for the HIV and TB antibodies on the epoxy slides ranged from 0.000954 to 250 µg/mL, where a 64-fold serial dilution was performed. The concentration range for the primary antibodies selected for this study is similar to what is commonly found in human blood during HIV and TB natural infections [39,40]. From the results obtained, the lowest detection concentration of the primary antibodies against all antigens was determined to be 0.000954 µg/mL for both PBS- and human serum-diluted antibodies (Table 1). The highest fluorescence intensity and the brightest spots were observed at the highest concentration of 250 µg/mL for all antigen–antibody interactions, and this intensity decreased with decreasing antibody concentration (Figure 6). No fluorescence was observed on the negative control spots where PBS was spotted instead of antigens. Although the linearity of the antibody binding curve was observed to be unexpectedly better when antibodies were diluted in human serum than in PBS (Appendix A, Figure A1), in general comparable results (same limit of detection) were obtained in both cases. This implies that proteins found in human serum are not likely to interfere with our diagnostic technology antigen–antibody reactions. 

The limit of detection was determined to be the lowest concentration of the primary antibody to give a fluorescence signal after subtraction of the background fluorescence (fluorescence emitted by the negative control spot).

**Figure A2:**
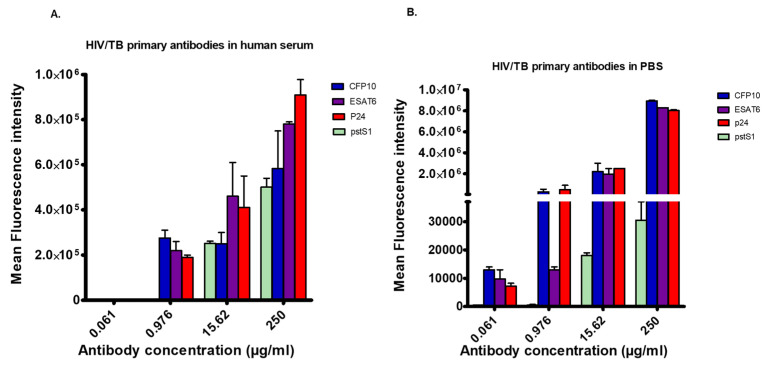
**Determination of antigen-antibody reaction sensitivity for the microtiter plates.** The concentrations of immobilized antigen and secondary antibodies were kept constant while that of the primary antibodies varied. (**A**) shows the fluorescence intensity obtained when HIV and TB antibodies were diluted in human serum, while (**B**) shows the intensity obtained when these antibodies were diluted in PBS. Error bars represent mean ± standard deviation (n = 4).

Since the use of high-binding plates for immunoassays is the gold standard [36,41], we replicated our microarray technology-based immunoassay (with primary antibodies diluted in PBS or human serum) in these plates to compare their performance. We observed that the limit of detection obtained from the high-binding plates was higher compared to the epoxy slides when the antibodies were diluted in either PBS or human serum (Figure 6, Appendix A, Figure A1 and Figure A2). To be precise, this limit in PBS was between 64 to 16,373-fold higher using the plates compared to the microarray slide, and the difference was statistically significant (*p* < 0.05) (Table 1). When the assay was performed in human serum, the limit of detection for the plates was lowered by 1023 to 16,373-fold compared to the microarray platform and the difference was statistically significant (*p* < 0.05). In conclusion, our diagnostic technology on microarray slides showed significantly higher sensitivity than when the same assay was performed on the high-binding plates which are commonly used. 

**Figure 7:**
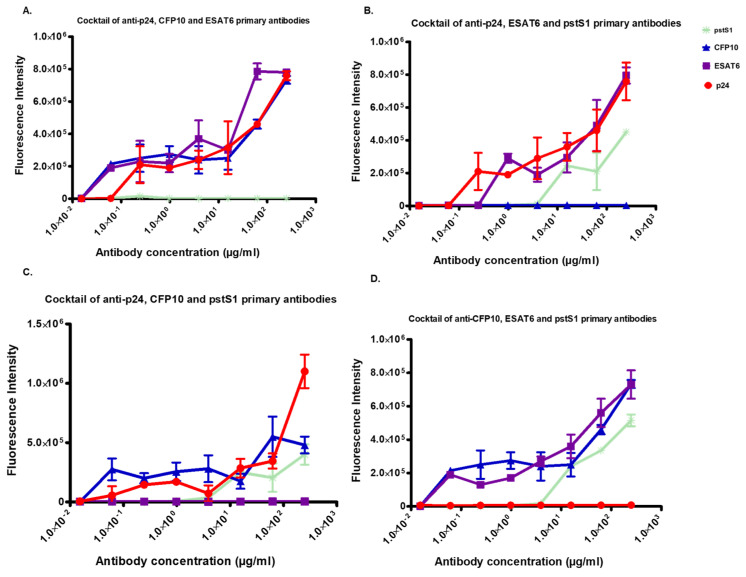
**Microarray plates’ antigen-antibody reaction specificity in human serum.** Immobilized antigens were incubated with cocktails of primary antibodies, missing one antibody at a time. Antigens and secondary antibody concentrations were kept constant, while primary antibodies were serially diluted. (**A**) A line graph representing the fluorescence intensity obtained when immobilized antigens were incubated in a cocktail of primary antibodies missing the anti-pstS1 antibody; (**B**–**D**) graphs for anti-CFP10 or anti-ESAT6 or anti-p24, respectively. Data represent mean ± standard deviation (n = 4).

**Figure A3:**
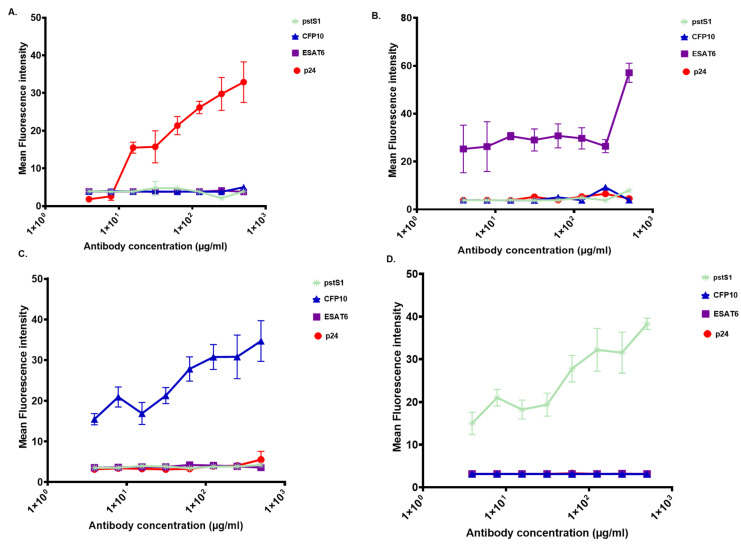
**Alternative set-up of Microarray plates’ antigen–antibody reaction specificity in human serum.** The immobilized antigens were incubated with the anti-p24 antibody (**A**), anti-ESAT6 antibody (**B**), anti-CFP10 antibody (**C**) or anti-pstS1 antibody (**D**) only, before the addition of the Alexa fluor 488-labelled secondary antibody. Data represent mean ± standard deviation (n = 3).

Lastly, to determine the specificity of our diagnostic technology, i.e., the absence of cross-reactivity, microarray slides containing all the antigens were incubated with a human serum cocktail of the primary antibodies, missing one antibody at a time. This was done to see if the antigen corresponding to the absent antibody would bind to the remaining primary antibodies in the cocktail. In another variation of this experiment, we incubated the four antigen-coated microarray slides with only one antibody at a time to see if this antibody would react with the other antigens besides its own. In either experimental set-up, we observed no cross-reactivity between the antigens and antibodies (Figure 7 and Appendix A, Figure A3). In all, these data suggest that our microarray-based technology is likely to perform with high specificity. 

## 4. Discussion

In this study, we sought to develop a multiplex technology for the detection of HIV and TB co-infection. Here, epoxy functionalized slides were used to immobilize HIV-1 p24 as well as *M.tb* antigens CFP10, ESAT6 and pstS1 using covalent chemistry, and these were used to capture their respective antibodies in solution. The sensitivity and specificity of this multiplex microarray-based antibody–antigen reaction was then studied and compared to the gold standard platform, i.e., high-binding 96-well plates. We found that the antigen–antibody reactions on the microarray slide were significantly more specific and sensitive compared to results obtained in high-binding plates. In fact, the limit of detection of the primary antibodies using our microarray-based diagnostic technology was on average about 4690-fold lower compared to the plates. We have also shown that the microarray technology in this study is stable at different pH and temperature conditions, as well as able to be stored dry for at least three months. These findings suggest that our microarray-based technology has potential for use to diagnose HIV and TB co-infection. Currently it is likely to be the first to do so, and the immunoassay conducted using this platform will probably perform better than when compared to commercially available kits that use the high-binding 96-well plates. These findings support further development of this technology for application in HIV and TB co-infection diagnostic.

The fact that the human serum we used was spiked with HIV and TB antibodies at a concentration that is commonly found in infected individuals [39,40] and that we were able to detect these antibodies using our microarray technology suggests that this technology is likely to work when actual human samples are used. Previous investigators have also spiked the analyte in human serum and found that the results could be replicated when actual human samples were used [42,43,44]. Furthermore, the use of human serum mimicked in vivo conditions where the HIV and TB antibodies could be present in the presence of other human proteins. The data obtained were compared to that of gold standard high-binding plates, and we found that the slides were more sensitive, with the average limit of detection determined to be 0.000954 µg/mL for antibodies against the four antigens, while for the plates this limit was on average 4.637 µg/mL. To explain the striking difference of the results obtained from these two platforms, it is important to note that on the slides the antigens are immobilized using a covalent bond while on the high-binding plates they are immobilized by adsorption using van der Waals, hydrophobic and/or electrostatic interactions [45]. Since the covalent bond is stronger than the van der Waals, hydrophobic and/or electrostatic interactions [46,47], the chance of some of the immobilized antigens being lost, for example by washing out during the assay, is greatly reduced on the slides compared to the plates. It is also possible that the high sensitivity of the microarray technology compared to the high-binding plate was due to the instrument used for the detection of fluorescence, as the slides were read using the cytation3 multimode reader while for the plates we used the plate reader DTX 880 Multimode detector. Nonetheless, the fact that we observed similar sensitivity when comparing PBS and human serum results for either the microarray slide or the microtiter plate indicates that the presence of other human proteins did not interfere with the antigen–antibody reaction on these two platforms. The similarity in this particular characteristic between the microarray slides and the high-binding plates was somewhat expected, because the interaction between the antigen–antibody does not rely on the surface chemistry of the microarray slides or high-binding plates as long as the immobilized antigen is in the correct conformation. Lastly, we did not observe any cross-reactivity between the antigen–antibody reaction on the microarray slides. This is consistent with numerous studies that have shown the high specificity of this reaction [48,49,50]. Also, the high specificity of protein–protein interactions on the microarray slide has been previously described; for example, Koukouvinos et al. studied the cross-reactivity of mycotoxins in a protein microarray and found that the antibody–mycotoxin reactions were highly specific [51]. 

The above being said, similar ELISA-based technologies, such as the electrochemical impedimetric ESAT6 immunosensor [52], gold-nanoparticle-based lateral flow TB immunoassay [53] and HIV p24 detection using combined immunoprecipitation and digital ELISA [54], have generally reported limits of detection less than ours of 0.000954 µg/mL (Table A1); however, our assay still has the main advantage of being able to detect HIV and TB at the same time. In addition, the combination of the selected three TB antigens is likely to enable our assay to detect active TB [32,33,34,35]. 

Since proteins have three-dimensional structures and can easily lose their reactivity due to factors such as pH and temperature, they are prone to denaturation during printing and immobilization if the conditions are not ideal [55]. Our data suggest that the proteins captured on our microarray slides are likely to remain stable at room temperature and neutral pH. This is critical, as routine laboratory diagnostics are commonly conducted at room temperature [56,57,58] and human blood has a pH of 7.4 [59,60]. Also, the fact that storage in dry conditions did not affect the performance of our microarray slide indicates that they can be stored without special requirements, which is very important, especially for resource-poor settings. Here, it is worth noting that HIV and TB co-infection is a bigger problem in developing countries, which often have limited resources [61,62]. Lastly, we tested the shelf-life of our technology for three months, and during this period we observed that fluorescence could still be obtained on all the antigens spots printed on the slide even on day 90. Thus, it is possible the actual shelf-life of this diagnostic technology is longer than three months, which could offer more advantage in resource constrained settings. However, this must be proved experimentally by conducting experiments beyond the time frames measured in this study. 

For coating the microarray slide, we selected epoxy as the functional group, as it interacts with the amine group at the N-terminus of the protein for immobilization [10,63]. This ensured that the immobilized protein is free to interact with its target [10,63]. Yousefi et al. showed that the epoxy functional group performed better as a microarray slide coating agent when compared to other functional groups [30]. Thus, it is unlikely that different functional groups would have performed better than the epoxy group used in this study. However, this study can still be replicated in future with these functionalized groups to compare the performance of the assay.

As for current limitations of the study, we think the data presented here can be complemented by those using actual human blood samples of HIV and TB co-infected patients. This being said, we are also aware that many investigators in the past used human serum spiked with antibodies, the same as we did here, for validation of technologies meant to detect biomarkers in human blood samples [64,65,66,67,68]. In addition, a thorough comparison with other diagnostic methods for active TB such as *M.tb* culture or HIV PCR and Western blotting will be very useful in assessing our novel technology performance. Lastly, as stated above, a longer storage study as well as a wider range of pH and temperature studies could be of benefit for further development of this technology.

In conclusion, we have shown that HIV-1 p24 as well as *M.tb* CFP10, ESAT6 and pstS1 antigens can successfully be conjugated or printed on epoxy-coated glass slides under different pH and temperature conditions. We have also shown that the immobilized antigens on the slides can be stored for at least three months without compromising their function. Most importantly, these antigens were able to capture their complementary primary antibodies in human serum, and the multiplex microarray diagnostic technology showed high specificity and sensitivity when compared to immunoassay performed on high-binding plates. Thus, this is a promising diagnostic technology that requires further development for the detection of HIV and TB co-infection. 

## Figures and Tables

**Table 1:** A head-to-head comparison of sensitivity between the epoxy-coated glass slide and high-binding plates.

Primary Antibody	Limit of Detection Concentration (µg/mL)			
	Primary Antibodies Diluted in PBS		Primary Antibodies Diluted in Human Serum	
	Epoxy-Coated Glass Slides	Polystyrene High-Binding Microtiter Plates	Epoxy-Coated Glass Slides	Polystyrene High-Binding Microtiter Plates
Anti-p24	0.000954	0.061	0.000954	0.9765
Anti-ESAT6	0.000954	0.06138	0.000954	0.9765
Anti-CFP10	0.000954	0.06138	0.000954	0.9765
Anti-pstS1	0.000954	15.62	0.000954	15.62

## Data Availability

The data sets used and/or analyzed during the present study are available from the corresponding author on request.

## Appendix A

**Table A1:** Head-to-head comparison of the multiplex HIV/TB microarray technology reported here and ELISA-based technologies in the literature for the detection of HIV or TB.

Method	Used Material	Target Matrix	Limit of Detection (LOD)
Multiplex HIV/TB microarray technology	-CFP10, ESAT6, pstS1 and p24 antigens and primary antibodies -Alexa fluor-conjugated secondary antibodies for fluorescence detection	-Human serum	-CFP10, ESAT6, pstS1 and p24 antibodies LOD of 0.000954 µg/mL
Electrochemical impedimetric ESAT-6 immunosensor [52]	-ESAT6 proteins and monoclonal antibodies -Label free (uses Ultrasensitive electrochemical immunosensors)	-Mice serum samples	-ESAT 6 protein LOD of 7.0 × 10^−6^ µg/ml
Gold Nanoparticle (AuNps)-based Lateral flow TB immunoassay [53]	-ESAT6 and CFP10 antibodies and antigens -AuNps-conjugated antibodies for colorimetric labelling	-Non-sputum samples	-ESAT6 antigen LOD of 0.0000625 µg/mL -CFP10 antigen LOD of 0.00769 µg/ml
HIV p24 detection using combined immunoprecipitation and digital ELISA [54]	-p24 antigen and p24 monoclonal antibodies -Biotin-labelled anti-p24 antibody	-Cell lysate	-p24 protein LOD of 5.0 × 10^−9^ µg/mL

## References

[1] UNIAIDS Global HIV & AIDS Statistics—Fact Sheet. 2021, pp. 1–7. https://www.unaids.org/en/resources/fact-sheet.

[2] Antony B., Tiewsoh J.B.A., Boloor R. (2020). HIV-TB co-infection with clinical presentation, diagnosis, treatment, outcome and its relation to CD4 count, a cross-sectional study in a tertiary care hospital in coastal Karnataka. J. Fam. Med. Prim. Care.

[3] Ríos-Hincapié C.Y., Rojas M., López M., Porras A., Luque R., Pelissari D.M., López L., Rueda Z.V. (2020). Delays in HIV and TB diagnosis and treatment initiation in co-infected patients in Colombia. Int. J. STD AIDS.

[4] Dewan R., Anuradha S., Khanna A., Garg S., Singla S., Ish P., Agarwal S., Narayana H.A., Hanif M., Singh H. (2015). Role of cartridge-based nucleic acid amplification test (CBNAAT) for early diagnosis of pulmonary tuberculosis in HIV. Indian Acad. Clin. Med..

[5] Scott L., da Silva P., Boehme C.C., Stevens W., Gilpin C. (2017). Diagnosis of opportunistic infections: HIV co-infections-tuberculosis. Curr. Opin. Hiv Aids.

[6] Cheon S.A., Cho H.H., Kim J., Lee J., Kim H.-J., Park T.J. (2016). Recent tuberculosis diagnosis toward the end TB strategy. J. Microbiol. Methods.

[7] Gina P., Randall P.J., Muchinga T.E., Pooran A., Meldau R., Peter J.G., Dheda K. (2017). Early morning urine collection to improve urinary lateral flow LAM assay sensitivity in hospitalised patients with HIV-TB co-infection. BMC Infect. Dis..

[8] Ryu Y.J. (2015). Diagnosis of Pulmonary Tuberculosis: Recent Advances and Diagnostic Algorithms. Tuberc. Respir. Dis..

[9] Liu H., Schittny V., Nash M.A. (2019). Removal of a Conserved Disulfide Bond Does Not Compromise Mechanical Stability of a VHH Antibody Complex. Nano Lett..

[10] Sauer U. (2017). Analytical Protein Microarrays: Advancements Towards Clinical Applications. Sensors.

[11] Sutandy F.X.R., Qian J., Chen C.-S., Zhu H. (2013). Overview of Protein Microarrays. Curr. Protoc. Protein Sci..

[12] Templin M.F., Stoll D., Schrenk M., Traub P.C., Vöhringer C.F., Joos T.O. (2002). Protein microarray technology. Drug Discov. Today.

[13] Haab B.B. (2005). Antibody Arrays in Cancer Research. Mol. Cell. Proteom..

[14] Seidel M., Niessner R. (2008). Automated analytical microarrays: A critical review. Anal. Bioanal. Chem..

[15] Chen Z.-A., Sun Y.-F., Wang Q.-X., Ma H.-H., Ma Z.-Z., Yang C.-J. (2021). Integrated Analysis of Multiple Microarray Studies to Identify Novel Gene Signatures in Ulcerative Colitis. Front. Genet..

[16] Govarthanan K., Gupta P.K., Ramasamy D., Kumar P., Mahadevan S., Verma R.S. (2019). DNA methylation microarray uncovers a permissive methylome for cardiomyocyte differentiation in human mesenchymal stem cells. Genomics.

[17] Lin X.M., Chen Y.H. (2018). Identification of Potentially Functional CircRNA-miRNA-mRNA Regulatory Network in Hepatocellular Carcinoma by Integrated Microarray Analysis. Med. Sci. Monit. Basic Res..

[18] Wang S., Yan R., Wang B., Meng P., Tan W., Guo X. (2019). The Functional Analysis of Selenium-Related Genes and Magnesium-Related Genes in the Gene Expression Profile Microarray in the Peripheral Blood Mononuclear Cells of Keshan Disease. Biol. Trace Elem. Res..

[19] Zhang H., Klose A.M., Miller B.L. (2021). Label-Free, Multiplex Glycan Microarray Biosensor for Influenza Virus Detection. Bioconjugate Chem..

[20] Zhang H., Liu X., Zhang C., Xu Y., Su J., Lu X., Shi J., Wang L., Landry M.P., Zhu Y. (2020). A DNA tetrahedral structure-mediated ultrasensitive fluorescent microarray platform for nucleic acid test. Sens. Actuators B Chem..

[21] Wu T., Li Y., Liang X., Liu X., Tang M. (2021). Identification of potential circRNA-miRNA-mRNA regulatory networks in response to graphene quantum dots in microglia by microarray analysis. Ecotoxicol. Environ. Saf..

[22] Ruano-Gallego D., García-Villadangos M., Moreno-Paz M., Gómez-Elvira J., Postigo M., Simón-Sacristán M., Reyburn H.T., Carolis C., Rodrigo N., Codeseira Y.B. (2021). A multiplex antigen microarray for simultaneous IgG and IgM detection against SARS-CoV-2 reveals higher seroprevalence than reported. Microb. Biotechnol..

[23] Khan S., Nakajima R., Jain A., De Assis R.R., Jasinskas A., Obiero J.M., Adenaiye O., Tai S., Hong F., Milton D.K. (2020). Analysis of Serologic Cross-Reactivity Between Common Human Coronaviruses and SARS-CoV-2 Using Coronavirus Antigen Microarray. bioRxiv.

[24] Pohlmann C., Elssner T. (2020). Multiplex Immunoassay Techniques for On-Site Detection of Security Sensitive Toxins. Toxins.

[25] Fan Y., Duan X., Zhao M., Wei X., Wu J., Chen W., Liu P., Cheng W., Cheng Q., Ding S. (2020). High-sensitive and multiplex biosensing assay of NSCLC-derived exosomes via different recognition sites based on SPRi array. Biosens. Bioelectron..

[26] Cohen L., Walt D.R. (2018). Highly Sensitive and Multiplexed Protein Measurements. Chem. Rev..

[27] Rusmini F., Zhong Z., Feijen J. (2007). Protein Immobilization Strategies for Protein Biochips. Biomacromolecules.

[28] Jian M., Zhang H., Li X., Wang Z. (2021). Profiling of multiple matrix metalloproteinases activities in the progression of osteosarcoma by peptide microarray-based fluorescence assay on polymer brush coated zinc oxide nanorod substrate. Sens. Actuators B Chem..

[29] Shlyapnikov Y.M., Malakhova E.A., Shlyapnikova E.A. (2020). Improving Immunoassay Performance with Cleavable Blocking of Microarrays. Anal. Chem..

[30] Yousefi H., Su H.-M., Ali M., Filipe C.D.M., Didar T.F. (2018). Producing Covalent Microarrays of Amine-Conjugated DNA Probes on Various Functional Surfaces to Create Stable and Reliable Biosensors. Adv. Mater. Interfaces.

[31] Zhu C., Zhu X., Landry J.P., Cui Z., Li Q., Dang Y., Mi L., Zheng F., Fei Y. (2016). Developing an Efficient and General Strategy for Immobilization of Small Molecules onto Microarrays Using Isocyanate Chemistry. Sensors.

[32] Davidow A., Kanaujia G.V., Shi L., Kaviar J., Guo X., Sung N., Kaplan G., Menzies D., Gennaro M.L. (2005). Antibody Profiles Characteristic of *Mycobacterium tuberculosis* Infection State. Infect. Immun..

[33] de Araujo L.S., da Silva N.D.B.M., Leung J.A.M., Mello F.C.Q., Saad M.H.F. (2018). IgG subclasses’ response to a set of mycobacterial antigens in different stages of Mycobacterium tuberculosis infection. Tuberculosis.

[34] Khurshid S., Khalid R., Afzal M., Waheed Akhtar M. (2013). Truncation of PstS1 antigen of Mycobacterium tuberculosis improves diagnostic efficiency. Tuberculosis.

[35] Watson A., Li H., Ma B., Weiss R., Bendayan D., Abramovitz L., Ben-Shalom N., Mor M., Pinko E., Bar Oz M. (2021). Human antibodies targeting a Mycobacterium transporter protein mediate protection against tuberculosis. Nat. Commun..

[36] Jørgensen M.M., Sloth J.K., Bæk R. (2021). Optimization of High-Throughput Multiplexed Phenotyping of Extracellular Vesicles Performed in 96-Well Microtiter Plates. Polymers.

[37] Tsougeni K., Ellinas K., Koukouvinos G., Petrou P.S., Tserepi A., Kakabakos S.E., Gogolides E. (2018). Three-dimensional (3D) plasma micro-nanotextured slides for high performance biomolecule microarrays: Comparison with epoxy-silane coated glass slides. Colloids Surf. B Biointerfaces.

[38] Mujawar L.H., Moers A., Norde W., van Amerongen A. (2013). Rapid mastitis detection assay on porous nitrocellulose membrane slides. Anal. Bioanal. Chem..

[39] Raux M., Finkielsztejn L., Salmon-Ceron D., Bouchez H., Excler J., Dulioust E., Grouin J., Sicard D., Blondeau C., Mestecky J. (1999). Comparison of the Distribution of IgG and IgA Antibodies in Serum and Various Mucosal Fluids of HIV Type 1-Infected Subjects. AIDS Res. Hum. Retroviruses.

[40] Kimuda S.G., Andia-Biraro I., Egesa M., Bagaya B.S., Raynes J.G., Levin J., Elliott A.M., Cose S. (2017). Use of QuantiFERON(R)-TB Gold in-tube culture supernatants for measurement of antibody responses. PLoS ONE.

[41] Viitala S.M., Jääskeläinen A.J., Kelo E., Sirola H., Moilanen K., Suni J., Vaheri A., Vapalahti O., Närvänen A. (2012). Surface-activated microtiter-plate microarray for simultaneous CRP quantification and viral antibody detection. Diagn. Microbiol. Infect. Dis..

[42] Yeung D., Ciotti S., Purushothama S., Gharakhani E., Kuesters G., Schlain B., Shen C., Donaldson D., Mikulskis A. (2016). Evaluation of highly sensitive immunoassay technologies for quantitative measurements of sub-pg/mL levels of cytokines in human serum. J. Immunol. Methods.

[43] Wang W., Zhou H., Lin H., Roy S., Shaler T.A., Hill L.R., Norton S., Kumar P., Anderle M., Becker C.H. (2003). Quantification of Proteins and Metabolites by Mass Spectrometry without Isotopic Labeling or Spiked Standards. Anal. Chem..

[44] Toedter G., Hayden K., Wagner C., Brodmerkel C. (2008). Simultaneous Detection of Eight Analytes in Human Serum by Two Commercially Available Platforms for Multiplex Cytokine Analysis. Clin. Vaccine Immunol..

[45] Bhakta S.A., Evans E., Benavidez T.E., Garcia C.D. (2015). Protein adsorption onto nanomaterials for the development of biosensors and analytical devices: A review. Anal. Chim. Acta.

[46] Liu S., Rong C., Lu T., Hu H. (2018). Identifying Strong Covalent Interactions with Pauli Energy. J. Phys. Chem. A.

[47] Mallinson P.R., Smith G.T., Wilson C.C., Grech E., Wozniak K. (2003). From Weak Interactions to Covalent Bonds: A Continuum in the Complexes of 1,8-Bis(dimethylamino)naphthalene. J. Am. Chem. Soc..

[48] Kapingidza A.B., Kowal K., Chruszcz M. (2020). Antigen-Antibody Complexes. Subcell Biochem..

[49] Cervia C., Nilsson J., Zurbuchen Y., Valaperti A., Schreiner J., Wolfensberger A., Raeber M.E., Adamo S., Weigang S., Emmenegger M. (2021). Systemic and mucosal antibody responses specific to SARS-CoV-2 during mild versus severe COVID-19. J. Allergy Clin. Immunol..

[50] Van Regenmortel M.H. (2014). Specificity, polyspecificity, and heterospecificity of antibody-antigen recognition. J. Mol. Recognit..

[51] Koukouvinos G., Karachaliou C.-E., Kanioura A., Tsougeni K., Livaniou E., Kakabakos S.E., Petrou P.S. (2021). Fluorescence Enhancement on Silver-Plated Plasma Micro-Nanostructured 3D Polymeric Microarray Substrates for Multiplex Mycotoxin Detection. Processes.

[52] Xia Y., Zhu H., Liu R., Li X., Xie X., Zhao J., Li J., Jiao X., Yang Z., Chen X. (2023). Picogram level electrochemical impedimetric immunosensor for monitoring Mycobacterium tuberculosis based on specific and sensitive ESAT-6 monoclonal antibody. Talanta.

[53] Seele P.P., Dyan B., Skepu A., Maserumule C., Sibuyi N.R.S. (2023). Development of Gold-Nanoparticle-Based Lateral Flow Immunoassays for Rapid Detection of TB ESAT-6 and CFP-10. Biosensors.

[54] Wu G., Cheney C., Huang Q., Hazuda D.J., Howell B.J., Zuck P. (2021). Improved Detection of HIV Gag p24 Protein Using a Combined Immunoprecipitation and Digital ELISA Method. Front. Microbiol..

[55] Ellington A.A., Kullo I.J., Bailey K.R., Klee G.G. (2010). Antibody-Based Protein Multiplex Platforms: Technical and Operational Challenges. Clin. Chem..

[56] Ndlovu Z., Fajardo E., Mbofana E., Maparo T., Garone D., Metcalf C., Bygrave H., Kao K., Zinyowera S. (2018). Multidisease testing for HIV and TB using the GeneXpert platform: A feasibility study in rural Zimbabwe. PLoS ONE.

[57] Shah M., Variava E., Holmes C.B., Coppin A., Golub J.E., McCallum J., Wong M., Luke B., Martin D.J., Chaisson R.E. (2009). Diagnostic Accuracy of a Urine Lipoarabinomannan Test for Tuberculosis in Hospitalized Patients in a High HIV Prevalence Setting. J. Acquir. Immune Defic. Syndr..

[58] Lawn S.D., Brooks S.V., Kranzer K., Nicol M.P., Whitelaw A., Vogt M., Bekker L.-G., Wood R. (2011). Screening for HIV-Associated Tuberculosis and Rifampicin Resistance before Antiretroviral Therapy Using the Xpert MTB/RIF Assay: A Prospective Study. PLoS Med..

[59] Kellum J.A. (2000). Determinants of blood pH in health and disease. Crit. Care.

[60] Brunskill S., Thomas S., Whitmore E., McDonald C.P., Dorée C., Hopewell S., Staves J., Cardigan R., Murphy M.F. (2012). What Is the Maximum Time That a Unit of Red Blood Cells Can Be Safely Left Out of Controlled Temperature Storage?. Transfus. Med. Rev..

[61] Parsons L.M., Somoskövi A., Gutierrez C., Lee E., Paramasivan C.N., Abimiku A., Spector S., Roscigno G., Nkengasong J. (2011). Laboratory Diagnosis of Tuberculosis in Resource-Poor Countries: Challenges and Opportunities. Clin. Microbiol. Rev..

[62] Karim S.S.A., Churchyard G.J., Karim Q.A., Lawn S.D. (2009). Health in South Africa 3 HIV infection and tuberculosis in South Africa: An urgent need to escalate the public health response. Lancet.

[63] Krizkova S., Heger Z., Zalewska M., Moulick A., Adam V., Kizek R., Zhang T.-X., Zhu G.-Y., Lu B.-Y., Zhang C.-L. (2015). Nanotechnologies in protein microarrays. Nanomedicine.

[64] Pultar J., Sauer U., Domnanich P., Preininger C. (2009). Aptamer–antibody on-chip sandwich immunoassay for detection of CRP in spiked serum. Biosens. Bioelectron..

[65] Mytych D.T., La S., Barger T., Ferbas J., Swanson S.J. (2009). The development and validation of a sensitive, dual-flow cell, SPR-based biosensor immunoassay for the detection, semi-quantitation, and characterization of antibodies to darbepoetin alfa and epoetin alfa in human serum. J. Pharm. Biomed. Anal..

[66] Mohammadi S., Salimi A., Hamd-Ghadareh S., Fathi F., Soleimani F. (2018). A FRET immunosensor for sensitive detection of CA 15-3 tumor marker in human serum sample and breast cancer cells using antibody functionalized luminescent carbon-dots and AuNPs-dendrimer aptamer as donor-acceptor pair. Anal. Biochem..

[67] Maple L., Lathrop R., Bozich S., Harman W., Tacey R., Kelley M., Danilkovitch-Miagkova A. (2004). Development and validation of ELISA for Herceptin detection in human serum. J. Immunol. Methods.

[68] Albrecht C., Kaeppel N., Gauglitz G. (2008). Two immunoassay formats for fully automated CRP detection in human serum. Anal. Bioanal. Chem..

